# Functional Versatility of the CDK Inhibitor p57^Kip2^

**DOI:** 10.3389/fcell.2020.584590

**Published:** 2020-10-07

**Authors:** Justine Creff, Arnaud Besson

**Affiliations:** Centre National de la Recherche Scientifique, Laboratoire de Biologie Cellulaire et Moléculaire du Contrôle de la Prolifération, Centre de Biologie Intégrative, Université de Toulouse, Toulouse, France

**Keywords:** p57^Kip2^, CDK inhibitor, cell cycle, development, transcription, stem cells, *CDKN1C*

## Abstract

The cyclin/CDK inhibitor p57^Kip2^ belongs to the Cip/Kip family, with p21^Cip1^ and p27^Kip1^, and is the least studied member of the family. Unlike the other family members, p57^Kip2^ has a unique role during embryogenesis and is the only CDK inhibitor required for embryonic development. p57^Kip2^ is encoded by the imprinted gene *CDKN1C*, which is the gene most frequently silenced or mutated in the genetic disorder Beckwith–Wiedemann syndrome (BWS), characterized by multiple developmental anomalies. Although initially identified as a cell cycle inhibitor based on its homology to other Cip/Kip family proteins, multiple novel functions have been ascribed to p57^Kip2^ in recent years that participate in the control of various cellular processes, including apoptosis, migration and transcription. Here, we will review our current knowledge on p57^Kip2^ structure, regulation, and its diverse functions during development and homeostasis, as well as its potential implication in the development of various pathologies, including cancer.

## Introduction

The cell division cycle is the fundamental process by which a cell duplicates its cytoplasmic and nuclear contents and divides in two genetically identical daughter cells. Cell cycle progression is driven by specific combinations of heterodimeric cyclin/CDK (cyclin-dependent kinase) complexes that license progression from one cell cycle phase to the next. In these complexes, the CDK is the catalytic subunit that has serine/threonine kinase activity and the cyclin subunit allows CDK activation and determines substrate specificity (Malumbres and Barbacid, 2005; Suryadinata et al., 2010). These actors are finely regulated at the level of transcription, localization, post-translational modifications (mainly phosphorylation) and protein degradation. Another level of regulation is their association with inhibitory partners called CKIs (Cyclin-dependent Kinase Inhibitor). CKIs are divided in two families according to their structure, CDK binding specificity and evolutionary origin: the INK4 family and the Cip/Kip family (Sherr and Roberts, 1999; Besson et al., 2008; Suryadinata et al., 2010).

The INK4 family (Inhibitors of CDK4) is composed of four proteins, p16^INK4A^, p15^INK4B^, p18^INK4C^, and p19^INK4D^ that specifically bind to CDK4 and CDK6 and inhibit their interaction with D-type cyclins, thus preventing their activation (Sherr and Roberts, 1999; Besson et al., 2008). In contrast, members of the Cip/Kip family, p21^Cip1/Waf1^ (p21) (Harper et al., 1993), p27^Kip1^ (p27) (Polyak et al., 1994; Toyoshima and Hunter, 1994) and p57^Kip2^ (p57) (Lee et al., 1995; Matsuoka et al., 1995) bind to both CDK and cyclin subunits and have the ability to inhibit all cyclin/CDK complexes (Sherr and Roberts, 1999; Besson et al., 2008). Cip/Kip proteins share a conserved cyclin/CDK interaction domain in their N-terminal part, but diverge in their C-termini, suggesting that they play specific roles (Yoon et al., 2012). By inhibiting CDK activity, CKIs block proliferation and they are considered as tumor suppressor.

More recently, the characterization of the CKI interaction networks indicates that Cip/Kip family members plays multiple functions in the cell and that their roles are not restricted to cell cycle control but also extends to the regulation of other cellular processes (Besson et al., 2008).

In this review, we focus on the least studied Cip/Kip family member, p57, and give an overview of its regulation, multiple functions in physiology and implication in pathology.

## The *CDKN1C* Gene

p57 is encoded by *CDKN1C*, located in the telomeric region of chromosome 11 at 11p15.5 in human (Matsuoka et al., 1995), and in the distal region of chromosome 7 in mice (Hatada and Mukai, 1995). *CDKN1C* contains four exons and three introns and p57 is encoded only from exons 2 and 3 (Tokino et al., 1996).

**FIGURE 1:**
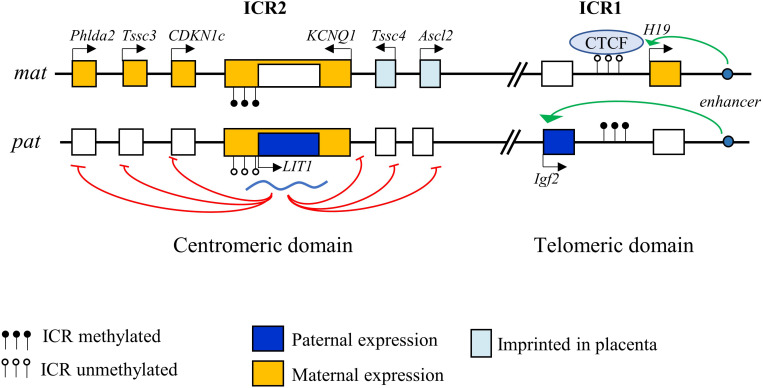
Imprinting control of the 11p15.5 locus. The 11p15.5 locus is organized in two domains, telomeric and centromeric, each controlled by an imprinting control region (ICR). Imprinting of the telomeric region is controlled by ICR1, which allows repression of *H19* and expression of *IGF2* on the paternal allele, and reciprocal expression on the maternal allele. ICR2 controls imprinting of the centromeric domain. Methylation of ICR2 leads to the suppression of the LIT1 antisense RNA of the *KCNQ1OT1* locus, allowing the expression of p57 from the maternal allele.

*CDKN1C* is a paternally imprinted gene, with preferential expression from the maternal allele (Matsuoka et al., 1996). The 11p15.5 locus contains a cluster of genes submitted to genomic imprinting. Parental or genomic imprinting is a process required for normal embryonic development that involves epigenetic modifications of a gene causing its monoallelic expression in a parental-dependent manner (Monk et al., 2019). Imprinted genes are commonly found in clusters, which contain imprinting control regions (ICRs) that are enriched in CpG islands and are differentially methylated on each allele. These regions also often include non-coding antisense RNAs that are essential for maintaining imprinting (Reik and Walter, 2001). Interestingly, the region homologous to 11p15.5 on murine chromosome 7 has the same gene cluster arrangement, highlighting the importance of this organization for the regulation of these genes. This locus covers about 1 Mb and is organized in two domains controlled by two regulatory centers, ICR1 and ICR2 (Figure 1).

ICR1 in the telomeric domain controls the reciprocal expression of *H19* (maternal expression) and *IGF2* (paternal expression). The expression of these two genes depends on the same pair of enhancers whose access is regulated by ICR1 (Leighton et al., 1995). On the maternal allele, unmethylated ICR1 is bound by CTCF (CCCTC-binding factor), a zinc-finger protein with insulating activity, preventing the interaction of the enhancers with the *IGF2* promoter, but allowing activation of the *H19* promoter, which is expressed. Conversely, on the paternal allele, CTCF does not recognize the methylated ICR1, allowing the interaction of the enhancers with the *IGF2* promoter and its expression, while *H19* is repressed (Hark et al., 2000; Eggermann et al., 2014b).

The centromeric domain is approximately 800 kb in size and contains several ubiquitously imprinted genes, including *KCNQ1, KCNQ1OT1* (*KCNQ1 Opposite strand/antisense transcript 1*, a.k.a. *LIT1* [*Long QT intron transcript 1*]), *CDKN1C*, *SLC22A18*, and *PHLDA2*, whose expression is regulated by ICR2. ICR2, located in intron 10 of *KCNQ1* corresponding to the *KCNQ1OT1* promoter, is methylated on the maternal allele, which represses KCNQ1OT1 expression and thereby allows maternal expression of all the other genes of the domain. Conversely, ICR2 is unmethylated on the paternal allele, allowing KCNQ1OT1 expression. KCNQ1OT1 is a non-coding antisense RNA that represses in cis the other genes of the domain (Fitzpatrick et al., 2002; Eggermann et al., 2014b) (Figure 1). KCNQ1OT1 directly binds to chromatin at the promoters of the imprinted genes and recruits histone methyltransferase complexes such as EZH2 (Enhancer of Zeste 2 Polycomb Repressive Complex 2 Subunit) and EHMT2 (Euchromatic Histone Lysine Methyltransferase 2), resulting in a repressive chromatin state (Pandey et al., 2008). In addition, KCNQ1OT1 recruits DNMT1 (DNA methyltransferase 1) to these promoters, causing their hypermethylation and reinforcing paternal repression (Mohammad et al., 2010). Imprinting defects in this locus are one of the most frequent cause of development of several syndromes (see below). Some genes in this locus are imprinted only in the placenta in mice (*Ascl2*, *CD81*, and *Tssc4*) (Shmela and Gicquel, 2013).

## Regulation of *CDKN1C* Expression

p57 expression is finely regulated by many signals and transcription factors. *CDKN1C* promoter analysis revealed the presence of several consensus sites for various transcription factors, including several SP1 (Stimulatory protein-1) sites, a Glucocorticoid response element (GRE) site, and a TATA box. The transcription factors E47, E2F1 and EGR1 (Early growth response gene 1), Tcf4, Hif1-α, as well as MyoD, via a p73 dependent mechanism, all promote p57 expression (Samuelsson et al., 1999; Rothschild et al., 2006; Vaccarello et al., 2006; Ma and Cress, 2007; Roeb et al., 2007; Wierenga et al., 2014). Stimulation with glucocorticoids or BMPs also induce p57 expression (Samuelsson et al., 1999; Gosselet et al., 2007; Shi and Liu, 2011; Zhang et al., 2015). Conversely, p57 transcription is repressed by the Notch effectors Hes1 and Herp2, as well as Id2 and PAX3-FOXO1 (Rothschild et al., 2006; Jia et al., 2007; Roeb et al., 2007; Riccio et al., 2008). TGF-β signals have antagonistic roles depending on cell type. Indeed, the TGF-β/Smad pathway stimulates p57 expression in hematopoietic stem cells, whereas it induces its degradation in osteoblasts (Urano et al., 1999; Scandura et al., 2004). The *CDKN1C* promoter is strongly regulated by methylation on the numerous CpG islands located upstream and downstream of the transcription start site, which appears to play a critical role in mediating p57 silencing in cancers (see below).

In carcinomas, multiple micro-RNAs have been shown to repress p57 expression. For example, p57 is targeted by miR221/222 in gastric, lung and hepatocellular cancers, promoting cell proliferation and tumor growth (Fornari et al., 2008; Kim et al., 2009; Wang et al., 2013). Similarly, p57 expression is down-regulated by miR25 in glioma and gastric cancer (Kim et al., 2009; Zhang et al., 2015) and by miR21 in prostate cancer (Mishra et al., 2014). In the context of stem cells, p57 is regulated by miR92. In human embryonic stem cells, miR92-b targets p57, promoting G_1_/S transition and stem cell proliferation (Sengupta et al., 2009). Conversely, in pancreatic cancer p57 is targeted by miR92-a, and miR92-a is downregulated in chemoresistant cancer stem cells, leading to p57 upregulation, which promotes cancer stem cell quiescence and treatment resistance (Cioffi et al., 2015). Recently, Long Non-Coding RNAs (lncRNAs) other than KCNQ1OT1 have also been identified as regulators of p57 expression. LncRNAs may regulate gene expression by several mechanisms, either acting as scaffolds, guides or by interacting with chromatin modifying proteins (Fatica and Bozzoni, 2014). Several lncRNAs overexpressed in cancer, notably SNHG17 in colorectal and gastric cancer, SH3PXD2A-AS1 in colorectal cancer, or LncRNA00511 in non-small cell lung carcinoma, were shown to downregulate p57 via their interaction with EZH2, the catalytic subunit of the Polycomb Repressive Complex 2 (PRC2), a methyltransferase that causes chromatin condensation (Sun et al., 2016; Ma et al., 2017, 2018; Zhang et al., 2019a). Inversely, LINC00628 is downregulated in colorectal cancer, which is associated with poor prognosis, and its interaction with EZH2 leads to p57 upregulation (Zhang et al., 2020). Other mechanisms have been identified, for instance in gastric cancer, the LncRNA ARHGAP27P1 activates p57 expression through binding to JMJD3 (Jumonji-Domain containing 3), causing demethylation of the p57 promoter (Zhang et al., 2019b).

Over the past few years, RNA modifications were shown to play an important role in RNA stability and translation and it appears that aberrant modifications are involved in tumorigenesis (Chen et al., 2019). NSUN2 (NOP2/Sun RNA methyltransferase 2), the main enzyme catalyzing 5-methylcytosine (m^5^C) formation, is upregulated in gastric cancer and promote cell proliferation and tumorigenesis via m5C methylation of the p57 mRNA, repressing p57 expression (Mei et al., 2020).

Unlike other Cip/Kip family members, p57 exhibits a tissue-specific expression pattern with marked variations of expression from embryogenesis to adulthood. During embryogenesis, p57 is strongly expressed from E9.5 to birth, with peak expression at key stages of differentiation in each organ. p57 is present in all three embryonic germ layers (endoderm, mesoderm, and ectoderm), and is found in the majority of organs: cartilage, skeletal muscle, heart, nervous system, and parenchymal organs (intestine, pancreas, lungs, adrenals, thymus, gonad, and kidney) as well as in extra-embryonic tissues. After E13.5, its expression strongly decreases in most tissues, but persists in skeletal muscle, kidney, intestine, palate, and lens (Nagahama et al., 2001; Westbury et al., 2001). In adults, p57 is expressed in post-mitotic cells of a limited number of tissues (heart, brain, lungs, skeletal muscle, placenta, kidney, gonads, intestine, and more weakly in the liver and spleen), which distinguishes it from the ubiquitous expression of p27 and p21 (Lee et al., 1995; Matsuoka et al., 1995).

## p57 Structure and Regulation

**FIGURE 2:**
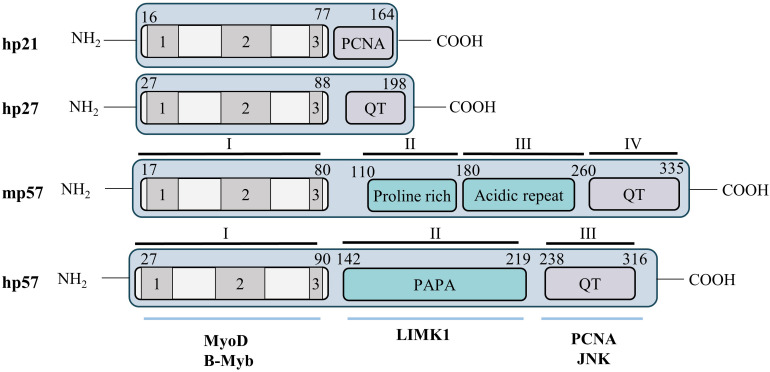
Structure of the p57^Kip2^ protein. Cip/Kip proteins are highly conserved in their N-terminal domain that mediates binding to cyclins and CDKs, but diverge in their C-terminal part. Nevertheless, the C-terminus of p57 presents some homology with p21 (PCNA binding domain) and p27 (QT motif). Murine and human p57 are conserved in their N- and C-terminal domains but the central domains (II and III) in mice are substituted by a unique PAPA domain in human (II). The N-terminal CDK binding/inhibitory domain (KID) of p57 is subdivided in three domains: a cyclin binding domain (1), a CDK binding domain (2) and a 3_10_ helix (3). The C-terminal QT domain (238–316) of p57 presents strong homology to the p27 QT motif at amino acids 302–316. It also contains three other conserved motifs: a PCNA binding domain (272–297) similar to p21, a nuclear localization signal (NLS, 278–281), and a CDK phosphorylation site (T310). Through its diverse domains p57 is able to interact with multiple partners involved in numerous cellular process, such as MyoD, b-Myb, LIMK1, PCNA, or JNK.

p57^Kip2^ is a protein of 316 amino acids in human and 335 in mice, with an apparent molecular weight of 57 kDa. The murine p57 protein is organized into four domains (Figure 2): domain I comprises the cyclin/CDK binding domain and has high homology to p21 and p27. Domain II is a Proline-rich region of 82 amino acids, followed by domain III of 107 amino acids rich in acidic residues (glutamic and aspartic acids). Finally, domain IV comprises a conserved motif called the QT domain presenting homology with p27 (QT motif) and p21 (PCNA binding domain). Human p57 is conserved in the C- and N-terminal parts, however, the central domains II and III are replaced by a single distinct domain rich in Proline/Alanine repeats, the PAPA domain (Figure 2) (Lee et al., 1995; Matsuoka et al., 1995). It is important to note that, like other Cip/Kip family proteins, p57 is an intrinsically unstructured protein that adopts a tertiary conformation only after binding to its different partners (Adkins and Lumb, 2002; Lacy et al., 2004).

The N-terminal domain I is necessary and sufficient for cyclin/CDK complex inhibition. This domain is subdivided in three regions: a cyclin binding domain, a CDK binding site and a 3_10_ helix. The 3_10_ helix is not required for binding to cyclin/CDKs but allows inhibition of Cyclin A/CDK2 and Cyclin E/CDK2 activity by inserting into the catalytic pocket of CDK2, thus blocking ATP hydrolysis, as reported for p27 (Russo et al., 1996; Hashimoto et al., 1998). This N-terminal domain also mediates the interaction with transcription factors such as b-Myb and MyoD (Reynaud et al., 2000; Joaquin and Watson, 2003). The PAPA domain is unique to p57 and may confer to p57 distinct functionalities from p21 and p27 via the interaction with different proteins. For example, this central region of p57 interacts with the kinase LIMK1, a Rho effector involved in regulation of actin polymerization (Yokoo et al., 2003). Finally, the C-terminal QT domain of p57 shares homology with the C-termini of both p27 (QT motif) and p21 (PCNA binding site). Importantly, the PCNA binding site of p57 was found to participate in mediating growth inhibition (Watanabe et al., 1998) and gain-of-function mutations in this domain were later discovered to cause the development of growth-restriction syndromes such as IMAGe (intrauterine growth restriction, metaphyseal dysplasia, adrenal hypoplasia congenita, and genital anomalies) and Silver Russell (see below) (Arboleda et al., 2012; Brioude et al., 2013b). The QT domain of p57 also mediates interaction with the stress-activated protein kinase JNK1 (Chang et al., 2003). A putative Nuclear Localization Sequence (NLS) consensus site has been identified, at the C-terminus of p57 by homology with p27 (Lee et al., 1995). Finally, the C-terminus of p57 contains a consensus site for CDK2 phosphorylation on Thr310 in human (Thr329 in mice) that allows degradation of p57, similarly to Thr187 on p27 (Kamura et al., 2003).

The degradation of p57 is mediated by the proteasome. Phosphorylation of Thr310 by cyclin E/CDK2 complexes creates a binding site for the F-box protein Skp2, leading to p57 ubiquitination by the Skp2-SCF (Skp1, Cullin, F-box) complex and to its degradation by the proteasome (Kamura et al., 2003). A second F-box protein, FBL12, has been involved in p57 degradation. In osteoblasts, TGFβ1 stimulates FBL12 expression that interacts with p57 phosphorylated on Thr310 and causes its proteasomal degradation independently of Skp2 (Kim et al., 2008).

## Animal Models of p57

Knockout of *Cdkn1c* in mice (p57^KO^) causes embryonic (approximately 10% of mutant embryos die between E13 and E16) and perinatal lethality, with less than 10% of animals surviving to adulthood (Yan et al., 1997; Zhang et al., 1997). p57^KO^ embryos display numerous developmental abnormalities that are mainly caused by defects in differentiation and increased apoptosis. For example, p57^KO^ mice frequently exhibit cleft palate, abdominal wall closure defects (umbilical hernia and omphalocele) associated with defective abdominal muscle development, truncation or shortening of the intestine, bone shortening and ossification delay due to defective chondrocyte differentiation, adrenal hyperplasia, renal dysplasia, and increased apoptosis in the lens (Table 1) (Yan et al., 1997; Zhang et al., 1997; Susaki et al., 2009). Consistent with *Cdkn1c* imprinting, animals inheriting a null allele from their mother (p57^+/–m^) exhibit a phenotype similar to homozygous p57^–/–^ animals. Importantly, a transgenic approach to insert a second *Cdkn1c* allele outside of the imprinted locus (i.e., two expressed copies of Cdkn1c) caused intrauterine growth restriction and embryonic lethality (Andrews et al., 2007). Thus, these studies have shown that unlike other CDK inhibitors, p57 is required for embryonic development and that expression of a single *Cdkn1c* allele is required.

**TABLE 1:** Summary of phenotypes observed in p57^KO^, p57^p27KI^, and p57^CK–^ mice.

p57^KO^	p57^p27KI^	p57^CK^^–^	Interpretation
• Neonatal death (90%)	Rescue	Phenocopy	CDK dependent
• Lens defect (60%)	Rescue	Phenocopy	CDK dependent
• Bone shortening (100%)	Rescue	Phenocopy	CDK dependent
• Bone shortening (100%)	Rescue	Phenocopy	CDK dependent
• Abdominal wall defect (95%)	Phenocopy	Phenocopy	CDK dependent, but p27 cannot rescue?
• Intestine shortening (30%)	Rescue	Rescue	CDK independent, common with p27?
• Kidney dysplasia (80%)	Phenocopy	Rescue	CDK independent
• Cleft palate (50%)	Rescue	Increase (76%)	CDK dependent and independent
• Adernal hyperplasia (70%)	Rescue 50%	Increase	CDK dependent and independent

Indeed, mice lacking p21 develop normally and are tumor free for the first 7 months (Deng et al., 1995), however aging mice (∼16 months) spontaneously develop tumors of hematopoietic, endothelial or epithelial origin (Martin-Caballero et al., 2001). Consistent with its CDK inhibitory function, p21 loss accelerates the development of Ras-induced mammary and salivary tumors (Adnane et al., 2000). Similarly, p27 loss does not cause overt developmental defects, however p27^KO^ mice are significantly larger and present multiple organ hyperplasia due to increased cell proliferation, and display retinal dysplasia and female sterility (Fero et al., 1996; Nakayama et al., 1996). p27 deficient animals spontaneously develop pituitary tumors within a few months and are dramatically more susceptible to carcinogenesis induced by genotoxic agents or oncogene activation (Fero et al., 1996, 1998; Nakayama et al., 1996). Moreover, p27 acts as a haploinsufficient tumor suppressor, as p27 heterozygote mice also display a predisposition to tumor development induced by carcinogens or irradiation (Fero et al., 1998) or in conjunction with inactivation of another tumor suppressor such as PTEN (Di Cristofano et al., 2001). Finally, an inactivating mutation in p27 was discovered in rats as the cause of MENX (Multiple Endocrine Neoplasia X) syndrome. This mutation is a tandem duplication of eight nucleotides causing a frameshift leading to rapid degradation of p27 and decreased p27 protein level (Pellegata et al., 2006). Subsequently, six germline mutations of p27 have been identified as the origin of a rare MEN syndrome in humans called MEN4 (Pellegata et al., 2006; Marinoni and Pellegata, 2011).

A number of functional redundancies and compensatory mechanisms between CKIs have been highlighted by the generation of double or triple mutants, as well as knock-in mutants (Tables 1, 2). Interestingly, double p27^–/–^/p57^KO^ or p21^–/–^/p57^KO^ knockout in mice exacerbate certain phenotypes of p57^KO^ mice. There is worsening of placental and lens defects, and increased embryonic lethality in p27^–/–^/p57^KO^ animals (Zhang et al., 1998). On the other hand, p21^–/–^/p57^KO^ double mutation aggravates skeletal defects, and causes the appearance of phenotypes not observed in the single mutants, such as impaired lung and skeletal muscle development (Zhang et al., 1999). Triple p21^–/–^/p27^–/–^/p57^KO^ knockout animals exhibit phenotypes similar to p27^–/–^/p57^KO^ mice and die between E11.5 and E15.5 (Tateishi et al., 2012) (Table 2) (for detailed review see, Ciemerych and Sicinski, 2005).

**TABLE 2:** Phenotypes associated with loss of Cip/Kip proteins in mice.

Genotypes	Phenotypes	References
p21^–/–^	Viable No major developmental anomalies	Deng et al., 1995

p27^–/–^	Viable Organomegaly, female sterility, increase body size	Nakayama et al., 1996 Fero et al., 1996

p57^–/–^	Lethal at birth Multiple developmental anomalies	Yan et al., 1997 Zhang et al., 1997

p21^–/–^ p27^–/–^	Viable, similar to p27^–/–^ with more pronounced ovarian hyperplasia	Tateishi et al., 2012 Jirawatnotai et al., 2003

p21^–/–^ p57^KO^	Lethal at birth, similar to p57^–/–^ with new phenotypes (impaired lung and skeletal muscle development)	Zhang et al., 1999

p27^–/–^ p57^KO^	Embryonic lethal (E12–E16.5), similar to p57^KO^ with more pronounced phenotypes (placenta, lens)	Zhang et al., 1998

p21^–/–^ p27^–/–^ p57^KO^	Embryonic lethal (E11.5–E15.5), similar to p27^–/–^ p57^KO^	Tateishi et al., 2012

In addition, a p57^p27KI^ knock-in mouse model, where the coding sequence of p27 was inserted in the p57 locus, revealed significant functional redundancy between p27 and p57 when compared with p57^KO^ mice, as p27 corrects certain phenotypes caused by the absence of p57, highlighting the importance of CDK inhibition by p57 during development (Table 1) (Susaki et al., 2009). Nevertheless, several phenotypes of p57^KO^ mice, such as kidney dysplasia, abdominal wall defects or adrenal hyperplasia, are not restored in p57^p27KI^ mutants, indicating that p57 plays specific functions during development (Table 1) (Susaki et al., 2009). Generation of another knock-in mutant, p57^CK–^, in which p57 no longer binds to cyclin/CDK complexes due to four point mutations, provided genetic evidence of cyclin/CDK independent functions of p57 during development (Duquesnes et al., 2016). Indeed, some phenotypes of p57^KO^ mice are completely absent in p57^CK–^ mice, such as kidney dysplasia and intestinal shortening (Table 1). Surprisingly, others phenotypes, including adrenal hyperplasia and cleft palate, were aggravated or more frequent in p57^CK–^ mice compared to p57^KO^, suggesting that in these tissues p57 plays both CDK dependent and independent roles (Table 1). A possible explanation for these phenotypes is that CDK-dependent degradation of the p57^CK–^ protein is partly defective, leading to increased levels of the mutant protein, which could potentially exert a dominant negative effect (Duquesnes et al., 2016).

## Functions of p57

### Canonical Function in Cell Cycle Regulation

The role of Cip/Kip family proteins as CDK inhibitor is well-established, and like p21 and p27, p57 causes a cell cycle arrest in G_1_ (Sherr and Roberts, 1999; Besson et al., 2008). p57 is able to bind and inhibit all cyclin/CDK complexes, however, with a lower affinity for cyclin B/CDK1 and cyclin D2/CDK6 complexes (Matsuoka et al., 1995). p57 mediates cyclin/CDK complex inhibition by blocking the substrate interaction domain on cyclins and by insertion into the catalytic pocket of the CDKs, thus preventing binding of ATP and catalytic activity (Russo et al., 1996). Like other Cip/Kip proteins, p57 promotes the assembly of Cyclin D1-CDK4/6 complexes, which may remain active (Labaer et al., 1997). Indeed, the CDK inhibitory activity of p27 and p21 is regulated by phosphorylation on Tyr88 and Tyr77, respectively, which relaxes the inhibitory conformation of the CKI, allowing partial CDK activation (Chu et al., 2007; Grimmler et al., 2007; James et al., 2008; Huang et al., 2015). This tyrosine is conserved in p57 (Tyr91), thus, a similar regulation of its CDK inhibitory activity by tyrosine kinases appears likely, although there is no direct evidence for this yet.

p57 can also inhibit the cell cycle via binding and inhibition of PCNA. Indeed, individual mutation of the CDK or PCNA binding site only results in partial loss of the growth inhibitory activity of p57 *in vitro*, whereas simultaneous loss of these two interactions completely abolishes the cell cycle inhibitory activity of p57 (Watanabe et al., 1998).

Finally, p57 also plays a role in trophoblast endoreplication. Endoreplication is a succession of G_1_ and S phases without intervening mitosis, leading to formation of giant polyploid cells. This phenomenon is observed during trophoblast differentiation to allow placenta implantation. p57 levels oscillate during endoreplication, decreasing before S phase entry and accumulating after S phase and in G_1_ (Hattori et al., 2000). p57^KO^ mice exhibit placental defects and it was shown *in vitro* that p57 is required to trigger mouse trophoblast endoreplication by inhibiting CDK1 activity (Ullah et al., 2008; Susaki et al., 2009).

### Non-canonical Functions of p57

In addition to cell cycle control, an increasing number of studies have described non-canonical functions of p57. The ability of p57 to regulate various cellular processes probably stems from its multi-domain structure and ability to interact with many protein partners. Indeed, a protein interactome has identified 183 direct potential partners of p57 involved in various cellular functions, including regulation of transcription, cytoskeleton and apoptosis (Duquesnes et al., 2016).

#### Cytoskeleton Regulation

All Cip/Kip family members have been shown to regulate cytoskeleton organization and cell migration by acting at different levels of the Rho/ROCK/LIMK/Cofilin pathway (Besson et al., 2004, 2008). *In vivo*, shRNA-mediated silencing of p57 delays neuronal migration in the cortex during development (Itoh et al., 2007) and p57 overexpression promotes radial migration of neural precursors (Tury et al., 2011). p57 is able to interact with LIMK1 via its unique central domain (Yokoo et al., 2003). LIMK1 is a serine/threonine kinase that promotes actin stress fiber assembly via phosphorylation of Cofilin, which inhibits its actin severing activity. LIMK1 is activated by the effectors of RhoA, ROCK1/2 (Rho-kinases) (Bernard, 2007). Binding of p57 to LIMK1 results in nuclear translocation and sequestration of LIMK1, which prevents Cofilin inhibition, resulting in decreased actin stress fiber formation (Yokoo et al., 2003). In agreement with these observations, p57 transfection into Schwann cells results in nuclear sequestration and inhibition of LIMK1, and a lack of Schwann cell differentiation (Heinen et al., 2008b). However, in HeLa cells the p57/LIMK1 interaction did not induce LIMK1 relocation in the nucleus, but increased its kinase activity, resulting in an increased number of stress fibers and decreased cell migration (Vlachos and Joseph, 2009). Similar findings were reported in hepatocellular carcinoma cells and decreased p57 levels in hepatocellular carcinoma samples correlated with the presence of metastases (Guo et al., 2015). In glioblastoma cells, p57 expression was also reported to inhibit cell migration and invasion (Sakai et al., 2004). Therefore, the role of p57 on cytoskeleton remodeling and migration appears to be mediated via its interaction with LIMK1 and to be dependent of the cellular context and subcellular location.

#### p57 and Apoptosis

Several phenotypes observed in p57^KO^ mice can be attributed to an increase of apoptosis caused by hyperproliferation and/or differentiation defects. Interestingly, p57 has the ability to modulate apoptosis by different mechanisms, and both pro and anti-apoptotic roles have been reported depending on the cellular context, the signaling pathway involved and the stress to which cells are submitted (Rossi and Antonangeli, 2015).

p57 protects against apoptosis indirectly by inhibiting CDKs. In response to stress, p38 phosphorylates p57 on Thr143, which increases its affinity for CDK2 and results in G_1_ cell cycle arrest (Joaquin et al., 2012). p38 or p57 deficient MEFs (Mouse Embryonic Fibroblasts) exhibit decreased viability in response to osmotic or oxidative stress and UV exposure (Joaquin et al., 2012). Overexpression of p57 in HEK293 or HeLa cells can also protect against apoptosis independently of CDK inhibition by regulating the JNK/SAPK signaling pathway. In this model, p57 interacts with JNK1 via its QT domain, preventing its interaction with c-Jun and thus inhibiting its kinase activity. Expression of the QT domain is sufficient to block JNK/SAPK mediated apoptosis in response to UVs or MEKK overexpression (Chang et al., 2003).

Conversely, in a HeLa Tet-On derived cell line that overexpresses p57 after induction, p57 potentiates apoptosis in response to different stresses. p57 sensitized cells to apoptosis induced by genotoxic agents such as staurosporine, cisplatin or etoposide, but had no effect on Fas-mediated apoptosis (Samuelsson et al., 2002; Vlachos et al., 2007). The pro-apoptotic effect of p57 in response to these drugs is CDK-independent and involves activation of the mitochondrial apoptosis pathway. In response to staurosporine, p57 relocates to mitochondrial membranes and promotes Bax activation and a decrease of mitochondrial membrane potential, resulting in cytochrome-c release in the cytosol and activation of caspases 9 and 3 (Vlachos et al., 2007). The specificity of the mitochondrial pathway was confirmed by overexpression of Bcl-2, which prevented the pro-apoptotic effect of p57 (Vlachos et al., 2007). The mechanism by which p57 activates the mitochondrial pathway is dependent on its ability to interact and stimulate LIMK1 activity. LIMK1-induced stabilization of actin fibers causes the displacement of hexokinase 1 (HK-1), a regulatory enzyme of the VDAC (Voltage-dependent Anion Channel) mitochondrial channel, allowing mitochondrial membrane depolarization and thus activation of the mitochondrial apoptotic pathway (Kavanagh et al., 2012). Finally, in H1299 non-small cell lung carcinoma cells, p57 transfection promoted p73β-mediated apoptosis in response to cisplatin, although the mechanism was not investigated (Gonzalez et al., 2005).

#### Regulation of Transcription by p57

Like other Cip/Kip family members, p57 can repress transcription indirectly via the inhibition of cyclin/CDK complexes, which prevents phosphorylation of Rb proteins and E2F transcription factors activation (Sherr and Roberts, 1999; Besson et al., 2008). p57 also negatively regulates the activity of RNA polymerase II via its interaction with E2F1. The interaction with E2F1 allows the recruitment of p57 to DNA, where it inhibits CDK7 and CDK9, resulting in decreased phosphorylation of the C-terminal domain (CTD) of RNA polymerase II and leading to a decrease of global transcription (Ma et al., 2010). In addition to this CDK-dependent regulation, p57 may also directly regulate the activity of transcription factors.

Indeed, p57 binds directly to b-Myb and inhibits its transcriptional activity independently of CDKs (Joaquin and Watson, 2003). p57 is also involved in regulating the stability of the bHLH transcription factor MyoD. p57 promotes MyoD stability and muscle differentiation by inhibiting CDK2, preventing MyoD phosphorylation by CDK2 on Ser200, which destabilizes the protein (Reynaud et al., 1999). p57 also stabilizes MyoD in a CDK-independent manner by interacting directly with MyoD via its N-terminal 3_10_ Helix region, protecting MyoD from degradation and promoting transactivation of muscle genes (Reynaud et al., 2000).

Similarly, p57 can interact directly with other bHLH transcription factors, including Ascl1 (Achaete-scute-like 1), NeuroD1 and Math2/NeuroD6 in neural progenitors. p57 then acts as a transcriptional repressor by directly inhibiting Ascl1 activity at target promoters, independently of CDKs (Joseph et al., 2009). Similarly, p57 can inhibit the transcriptional activity of Nurr1 independently of CDKs *in vitro*, and thus regulates dopaminergic neuron maturation (Joseph et al., 2003).

Finally, it was shown recently that p57 may also positively regulate transcription (Kullmann et al., 2020). p57 binds to and activates FHL2 (four-and-a-half LIM only protein 2), a multifunctional LIM domain only protein, which binds to and modulates the activity of several transcription factors, such as AP-1, β-catenin or the androgen receptor, by acting as a coactivator (Muller et al., 2000; Tran et al., 2016). FHL2 is inhibited by HDACs, and by binding to FHL2, p57 competes with HDAC1 and HDAC3 for FHL2 binding and prevents its inhibition (Kullmann et al., 2020).

Moreover, an interactome revealed that p57 has multiple partners involved in transcriptional regulation, including many transcription factors and HDAC7 (histone deacetylase 7), suggesting that p57 plays a major role in the regulation of transcription (Duquesnes et al., 2016).

#### p57 and Differentiation

p57 plays a key role in the differentiation of many cell types by inhibiting CDKs, promoting cell cycle exit, or via CDK-independent mechanisms. Several phenotypes observed in p57^KO^ mice are caused by a delay or defect of differentiation (Yan et al., 1997; Zhang et al., 1997).

Several studies have shown that p57 regulates various aspects of neurogenesis, notably by controlling cell differentiation in the central and peripheral nervous system. p57 is present in neural stem cells and controls their fate by promoting differentiation into astrocytes at the expense of the oligodendrocyte lineage (Jadasz et al., 2012). In the peripheral nervous system, p57 inhibits Schwann cell differentiation and its silencing by shRNA results in cell cycle exit, cell growth and differentiation, as well as an increase of myelin production (Heinen et al., 2008a). Similarly, in the central nervous system, p57 inhibits oligodendrocyte differentiation (Kremer et al., 2009). However, other reports found that p57 promotes differentiation of oligodendrocyte progenitors in rat primary cells *in vitro*, as well as *in vivo* in zebrafish (Park et al., 2005; Dugas et al., 2007). In the nucleus, p57 interacts and inhibits Ascl1, a transcription factor promoting oligodendrocyte differentiation (Nakatani et al., 2013; Gottle et al., 2015). During oligodendrocyte differentiation, p57 relocates to the cytoplasm, promoting the export and inhibition of the transcriptional repressor Hes5 and relieving Ascl1 inhibition, enhancing differentiation (Gottle et al., 2015). In this study, p57 regulated the localization of LIMK1 and CDK2, which contributed to oligodendrocyte differentiation (Gottle et al., 2015). Thus, the effect of p57 on oligodendrocyte differentiation appears dependent on its subcellular localization. In addition to these cell types, p57 is involved in cell cycle exit of neural progenitors and in the specification of amacrine retinal interneurons (Dyer and Cepko, 2000), as well as in the differentiation of dopaminergic neurons (Joseph et al., 2003). In the latter, the transcription factor Nurr1 up-regulates p57 expression, and in turn, p57 binds to and inhibits Nurr1 transcriptional activity, promoting differentiation (Joseph et al., 2003).

p21 and p57 cooperate and are required *in vivo* for terminal differentiation of skeletal muscle cells. p21^–/–^/p57^KO^ mice exhibit altered skeletal muscle differentiation due to increased proliferation, myoblast apoptosis, causing defects in myotube formation (Zhang et al., 1999). Expression of p57 increases during muscle differentiation and is induced indirectly by MyoD, via p73. In turn, p57 stabilizes MyoD in a positive feedback loop, promoting myogenesis (Vaccarello et al., 2006). MyoD can also directly stimulates p57 expression epigenetically by binding a negative regulatory *cis*-element, causing chromatin remodeling and lifting the inhibition of p57 expression (Busanello et al., 2012). The transcription factors Sp1 and Egr1 also participate in induction of p57 during myogenic differentiation (Figliola et al., 2008). In rhabdomyosarcomas, the chimeric protein PAX3-FOXO1 indirectly inhibits p57 transcription via destabilization of Egr1, which prevents myoblasts differentiation. Re-expressing p57 in these cells is sufficient to restore myogenic differentiation (Roeb et al., 2007).

p57 also participates in chondrocyte differentiation. Chondrocytes hypertrophic differentiation is required for ossification. Differentiated chondrocytes are characterized by high collagen X expression, strong alkaline phosphatase activity, and increased cell volume (Stewart et al., 2004). p57^KO^ mice exhibit bone shortening and an ossification delay caused by delayed chondrocyte differentiation associated with increased proliferation and decreased collagen X expression (Yan et al., 1997; Zhang et al., 1997). In chondrocytes, p57 expression is stimulated by C/EBPβ during differentiation, leading to cell cycle arrest (Hirata et al., 2009) and p57 also potentiates the induction of collagen X expression mediated by BMP2 (Stewart et al., 2004).

Finally, in several tissues, the Notch/Hes1 signaling pathway inhibits p57 expression to promote proliferation of progenitors and prevent cell cycle exit and thus precocious differentiation, notably in the intestine (Riccio et al., 2008), lens (Jia et al., 2007), and pancreas (Georgia et al., 2006). Indeed, loss of p57 expression in human is associated with hyperinsulinism during infancy due to increased production of β-islet cells (Kassem et al., 2001; Avrahami et al., 2014). Conversely, bi-allelic expression of p57 or gain-of-function mutations associated with the IMAGe syndrome lead to reduced β-islet cell number and predisposition to diabetes (Kerns et al., 2014; Asahara et al., 2015). In fact, p57 appears to be a target of choice to promote the regeneration of pancreatic β-islets (Avrahami et al., 2014; Ou et al., 2019).

#### p57 in the Regulation of Stem Cell Fate and Maintenance

Several studies have revealed a crucial role of p57 in maintaining quiescence of resident adult stem cells in multiple tissues.

p57 is required for hematopoietic stem cell quiescence and their maintenance. Indeed, in a conditional knockout model, p57 ablation in adult mice results in a decreased hematopoietic stem cell pool caused by an exit of quiescence state (G_0_) and induction of apoptosis (Matsumoto et al., 2011b). Reconstitution of the hematopoietic system after transplantation is also reduced in absence of p57 (Matsumoto et al., 2011b). These hematopoiesis defects are corrected by expression of p27 in the p57 locus (p57^p27KI^ knock-in mice) (Matsumoto et al., 2011b). In hematopoietic stem cells of the fetal liver, p57 loss is accompanied by an increase of p27 expression, nevertheless neither p21 nor p27 knockout causes any defect, confirming that p57 is the primary CKI required for hematopoietic stem cell maintenance (Zou et al., 2011). Consistently, TGF-β1 induces quiescence of hematopoietic stem cells and increases p57 levels (Yamazaki et al., 2009). Conversely, increased Skp2 expression, which induces p57 degradation, is necessary for cell cycle reentry of hematopoietic stem cells (Rodriguez et al., 2011). The role of p57 in controlling hematopoietic stem cell quiescence involves its interaction with the chaperone Hsc70, which results in cytoplasmic sequestration of Hsc70/Cyclin D1 complexes, inhibiting cell cycle entry. Similarly, p27 may also interact with Hsc70 to compensate for the loss of p57 (Zou et al., 2011).

In the lung, homeostasis and tissue regeneration are supported by resident pulmonary stem cells called BASCs (Bronchioalveolar Stem Cells) (Kim et al., 2005; Liu et al., 2019; Salwig et al., 2019). The self-renewal of these stem cells requires a precise regulation of p57 expression levels. Indeed, either loss or increase of p57 expression in BASCs causes a lack of self-renewal, leading to defective tissue regeneration (Zacharek et al., 2011). These results highlight the importance of p57 imprinting and monoallelic expression. In the lung, Bmi1 controls the expression of p57 and other imprinted genes, however, no overt methylation changes at individual promoters or ICRs were observed in absence of Bmi1, suggesting that Bmi1 regulates imprinted gene expression by another mechanism (Zacharek et al., 2011). How p57 controls BASC maintenance has not yet been investigated.

In the adult hippocampus, p57 is expressed in quiescent neural stem cells and absent in proliferative progenitors. The presence of p57 is critical for maintaining neural stem cell quiescence and while p57 ablation initially leads to an increase in stem cell proliferation and stimulates neurogenesis, it results over time in the exhaustion of the neural stem cell population and impairs neurogenesis in aged mice (Furutachi et al., 2013). Similarly, Nestin^Cre^-driven deletion of p57 in the developing brain causes a decrease of Pax2^+^ interneuron progenitor number due to massive p53-dependent apoptosis, resulting in hydrocephalus (Matsumoto et al., 2011a). In contrast, during cortical development, p57^KO^ embryos exhibit macrocephaly caused by an increased proliferation of neural progenitors and stem cells (Mairet-Coello et al., 2012). p57 appears to play a key role in timing cell cycle exit of specific neural progenitor populations, and p57 deregulation leads to abnormal development of specific neuron layers, especially during late neurogenesis (Mairet-Coello et al., 2012). A recent study using the MADM (mosaic analysis with double markers) system revealed that p57 regulates cortical neurogenesis by distinct mechanisms (Laukoter et al., 2020). p57 controls neural stem cell proliferation in a non-cell autonomous manner, with a complete knockout causing macrocephaly (Laukoter et al., 2020). However, conditional deletion of p57 in radial precursor (Emx1^+^ cells) causes microcephaly due to p53-mediated apoptosis, in this case p57 exerts a cell-autonomous growth-promoting function by promoting survival of maturing cortical projection neurons (Laukoter et al., 2020), consistent with the Nestin^Cre^*-*driven phenotype previously observed (Matsumoto et al., 2011a). Interestingly, there is some contribution of the paternal allele during mouse brain development since Nestin^Cre^*-driven* deletion of the paternal allele results in reduced adult brain size due to increased apoptosis of neural progenitors and a slight reduction of proliferation (Imaizumi et al., 2020). These findings are consistent with earlier observations that p57 exhibits biallelic expression in the developing brain (Matsuoka et al., 1996). Finally, p57 was recently involved in neural stem cell fate determination. In *Drosophila*, neural stem cells can enter a quiescence state either in G_0_ or G_2_ (25 and 75%, respectively) that determines their fate, as G_2_ blocked cells can be rapidly activated to divide in response to nutritional stimuli, while G_0_ cells respond more slowly (Otsuki and Brand, 2018). The choice between these two types of quiescence is determined at the embryonic stage and depends on the presence of the p57 homolog, Dacapo, which favors entry in the G_0_ quiescent state at the expense of the G_2_ one (Otsuki and Brand, 2019). Dacapo deletion leads to a change in the fate of the neural stem cells, which are then preferentially oriented toward a G_2_ quiescence state (Otsuki and Brand, 2019).

In the developing skeletal muscle, p57 inhibition is required for maintenance of certain stem/progenitor cells. Notch inhibits p57 expression in Pax3^+^/7^+^ muscle progenitors via Hes1, allowing amplification of the progenitor pool (Zalc et al., 2014). Loss of Notch signaling leads to an increase of p57 expression in muscle progenitors, which is associated with precocious differentiation and progenitor depletion (Zalc et al., 2014). In adult muscle, satellite cells, the resident stem cells, derive from Pax3^+^/7^+^ progenitors and are mobilized in case of damage to regenerate muscle fibers. Surprisingly, p57 is not expressed in quiescent satellite cells but is induced during their mobilization (Mademtzoglou et al., 2018). In addition, loss of p57 *in vivo* increases proliferation and self-renewal of progenitors and myoblasts at the expense of their differentiation, and in case of damage, p57 absence causes satellite cell depletion and delays muscle regeneration (Mademtzoglou et al., 2018).

Finally, the presence of p57 was reported in other quiescent adult tissue stem cells, such as hair follicle stem cells (Leishman et al., 2013) as well as in Mex3a^High^ quiescent intestinal stem cells (Barriga et al., 2017), suggesting that p57 plays a general role in the maintenance of resident stem cells.

## p57 in Human Pathologies

Deregulation of imprinting of the 11p15.5 locus, as well as loss of heterozygosity (LOH) or point mutations of *CDKN1C* are responsible for the development of several hereditary pathologies and may contribute to cancer development and progression. Alterations that cause loss of p57 function lead to the development of the Beckwith–Wiedemann syndrome, while p57 gain of function leads to the development of Silver-Russell and IMAGe syndromes.

### Beckwith–Wiedemann Syndrome

Beckwith–Wiedemann syndrome (BWS) (OMIM # 130650) is a rare genetic disorder with a prevalence of 1/13700 births, characterized by excessive growth, developmental anomalies and tumor predisposition during childhood. BWS has a highly variable presentation and patients usually display only a subset of phenotypes. Diagnosis is based on the presence of at least three major criteria (macrosomia, macroglossia, abdominal wall defects, hemihyperplasia, embryonal tumors, renal abnormalities, anterior ear lobe creases) or the association of two major criteria with at least one minor phenotype (neonatal hypoglycemia, characteristic facies, diastasis recti, cardiomegaly/structural cardiac anomalies…) (Weksberg et al., 2010). A molecular anomaly, of epigenetic or genetic origin, in the 11p15.5 region is identified in approximately 80% of BWS patients. Most epigenetic abnormalities are mosaic, meaning that only a fraction of the cells carry the molecular defect. The severity and penetrance of the phenotypes therefore depends on the underlying molecular mechanism and the percentage of mosaicism (Brioude et al., 2013a).

The majority of BWS patients present epigenetic abnormalities. Loss of ICR2 methylation on the maternal allele is the most common form and is observed in 50% of patients (Eggermann et al., 2014b). This anomaly results in biallelic expression of *KCNQ1OT1* and thus in loss of *CDKN1C* expression (Figure 1) (Eggermann et al., 2014b). Patients with anomalies of this centromeric region have a more severe phenotype and exhibit macroglossia and abdominal wall defects. Gain of methylation on ICR1 is observed in 5% of cases, resulting in biallelic expression of *IGF2*. Patients with this defect display a higher risk of developing tumors and organomegaly (Weksberg et al., 2010; Brioude et al., 2013a). Several genetic alterations are also responsible for BWS development. Among genetic anomalies, paternal isodisomies (UPD = Uniparental Disomy) are found in approximately 20% of patients. UPDs correspond to the duplication of the paternal 11p15.5 locus, with no maternal contribution for this region, thus leading to biallelic *IGF2* expression and loss of *CDKN1C* expression (Weksberg et al., 2010). Patients with UPDs have an increased risk of hemihyperplasia (Brioude et al., 2013a). Other chromosomal rearrangements have also been reported in rare (2–3%) cases, including extensive paternal duplications, carrying both ICR1 and ICR2 regions (1%), translocations/inversion (1%), which are usually transmitted by the mother, as well as ICR microdeletions (<1%) (Weksberg et al., 2010; Eggermann et al., 2014a). Finally, maternally inherited point mutations in *CDKN1C* are observed in 5% of sporadic cases and are responsible for the majority of familial cases (50%) of BWS (Eggermann et al., 2014b). Patients with *CDKN1C* mutations have particular phenotypes: almost all of them display abdominal wall defects (umbilical hernia or omphalocele) and have an increased incidence of cleft palate, genital anomalies and polydactyly (Romanelli et al., 2010). Mutations of *CDKN1C* described in BWS cover the entire coding region. They are mostly nonsense mutations leading to truncation of the protein or mutations causing a frameshift, which strongly alters the structure of the protein. Some missense mutations have also been described in the cyclin/CDK binding domain (Eggermann et al., 2014b; Brioude et al., 2015).

### Silver Russell and IMAGe Syndromes

In opposition to BWS, p57 gain of function mutations are associated with two very rare disorders, Silver-Russell syndrome (SRS) (OMIM # 180860) (1/100,000 birth) (Brioude et al., 2013b) and IMAGe syndrome (Intrauterine Growth Retardation, Metaphyseal dysplasia, Adrenal insufficiency, Genital abnormalities) (OMIM # 614732) (only a few dozen cases reported worldwide) (Arboleda et al., 2012). These pathologies cause opposite phenotypes to BWS and share a number of features, notably intrauterine growth retardation (Eggermann et al., 2014b).

The study of a familial form of IMAGe syndrome led to the identification of five missense mutations located in the PCNA binding domain of p57 (Arboleda et al., 2012). Expression of a transgene carrying these mutations in *Drosophila* caused decreased eye, wing and vascular network size, consistent with a gain of function in p57 (Arboleda et al., 2012). *In vitro*, these mutations were found to decrease binding to PCNA and to impair p57 ubiquitination, leading to p57 stabilization and inhibition of proliferation (Arboleda et al., 2012; Borges et al., 2015). Interestingly, mutation in DNA polymerase ε were recently identified in 15 cases of IMAGe syndrome (Logan et al., 2018). Given the impact of p57 mutations found in IMAGe on its interaction with PCNA, it would be interesting to investigate further their effect on PCNA function, DNA replication and S phase progression.

The most frequent molecular anomalies in SRS patients are loss of ICR1 methylation (60% of cases), which result in biallelic expression of *H19* and thus loss of *IGF2* expression. Chromosomal rearrangements with maternal duplications of the centromeric domain, containing ICR2, or of both domains have also been observed (Wakeling et al., 2017). Nevertheless, point mutations in *CDKN1C* causing a gain of function in p57 were recently found to cause SRS. Indeed, a gain of function mutation in the PCNA binding domain of p57 was identified in a familial form of SRS (Brioude et al., 2013b). Interestingly, the residue mutated (Arg279Leu) is also mutated in IMAGe syndrome (Arg279Pro). Tissue culture experiments suggest that this mutation in p57 does not affect the cell cycle but leads to stabilization of the protein, consistent with the ubiquitination defect previously observed in IMAGe syndrome (Brioude et al., 2013b).

### p57 and Cancer

Like other CKIs, p57 is a putative tumor suppressor. However, the lethality of p57^KO^ mice has so far prevented to test this formally *in vivo* and the involvement of p57 in cancers remains to be addressed in animal models. Nevertheless, several lines of evidence indicate that p57 plays a role in carcinogenesis. For instance, BWS patients are predisposed to tumor development (Weksberg et al., 2010) and decreased p57 expression is observed in many types of tumors, including in gastric, colorectal, pancreatic, pulmonary, and mammary carcinomas, as well as in leukemia (Li et al., 2003; Pateras et al., 2009; Borriello et al., 2011; Kavanagh and Joseph, 2011; Weis et al., 2015). Moreover, decreased p57 expression correlates with aggressiveness in several types of tumors and is associated with poor prognosis (Kavanagh and Joseph, 2011; Qiu et al., 2018). Interestingly, *CDKN1C* mutations are not frequently observed in cancers (Pateras et al., 2009). Loss of p57 expression in human carcinomas is caused predominantly by loss of heterozygosity of the 11p15.5 locus, methylation of the *CDKN1C* promoter, and histone methylations (Kikuchi et al., 2002; Pateras et al., 2006; Weis et al., 2015). In fact, loss of imprinting of KCNQ1OT1 has been observed in colorectal cancer samples, but did not necessarily correlated with p57 expression (Nakano et al., 2006). However, loss of p57 expression has been observed in colorectal cancer, mainly due to promoter hypermethylation (Kikuchi et al., 2002) and it was shown that the expression of the methyltransferase DNMT3a is strongly increased in colorectal tumors (Weis et al., 2015). Consistently, DNMT3a deletion inhibits tumor formation *in vivo* at least in part by increasing p57 expression (Weis et al., 2015). Moreover, several miRNAs and LncRNAs have also been shown to repress p57 in various cancers, as described earlier (Kavanagh and Joseph, 2011; Stampone et al., 2018). Finally, loss of p57 may also be due to increased degradation caused by Skp2 overexpression (Pateras et al., 2006).

## Conclusion

p57^Kip2^ has the particularity of being the only CDK inhibitor required for embryonic development. Since its discovery as a cyclin/CDK inhibitor, a growing number of studies have shown that p57 is a multifunctional protein involved in the regulation of other cellular processes such as cell migration, differentiation, apoptosis, transcriptional regulation or stem cell specification and fate. The generation of a p57^CK–^ knock-in mouse model has provided genetic evidence that some of its functions are independent of cyclins/CDKs during development, yet the underlying mechanisms remain largely unknown. How p57 is regulated also remains largely unclear. In view of its multiple functions, a better understanding of p57’s functions and regulation would improve our comprehension of normal development and of the etiology of several genetic disorders and cancers. The fact that p57 is subjected to paternal imprinting makes it particularly vulnerable to genetic alterations leading to loss (BWS, cancer) or gain (IMAGe and SRS) of function of the only active allele. In addition to complex developmental growth disorders, p57 down-regulation is associated with the development of cancers and its decreased expression is correlated with aggressiveness in several tumor types. For example, decreased p57 expression correlates with poor clinical outcome in breast cancer (Yang et al., 2009). Similarly, p57 levels are inversely related to tumor growth and cancer stage in non-small cell lung cancer, hepatocellular and pancreatic carcinomas and others (Ito et al., 2001a,b; Pateras et al., 2006). Interestingly, in colorectal cancer, p57 levels are increased in low grade adenomas and finally decrease in primary carcinomas (Li et al., 2003), perhaps reflecting a mechanism to initially limit tumor progression. Similarly, in Wilm’s tumor, loss of heterozygosity of the 11p15.5 locus is associated with the reactivation of the paternal allele of p57, possibly to limit cell proliferation and cancer progression (Overall et al., 1996; Taniguchi et al., 1997). To conclude, several lines of evidence indicate a prominent role for p57 during tumorigenesis and stem cell regulation, and a significant number of studies suggest that p57 is a pertinent prognostic marker and that it may hold therapeutic potential both for anticancer treatment and to manipulate tissue progenitor/stem cells for regenerative medicine.

## Author Contributions

All authors listed have made a substantial, direct and intellectual contribution to the work, and approved it for publication.

## Conflict of Interest

The authors declare that the research was conducted in the absence of any commercial or financial relationships that could be construed as a potential conflict of interest.
